# Novel genome polymorphisms in BCG vaccine strains and impact on efficacy

**DOI:** 10.1186/1471-2164-9-413

**Published:** 2008-09-15

**Authors:** Andrea S Leung, Vanessa Tran, Zuowei Wu, Xuping Yu, David C Alexander, George Fu Gao, Baoli Zhu, Jun Liu

**Affiliations:** 1 Department of Molecular Genetics, University of Toronto, 1 King's College Circle, Toronto, Ontario, Canada; 2 Joint Center for Microbial Genomics, Institute of Microbiology, Chinese Academy of Sciences, Beijing, PR China; 3 Department of Veterinary Medicine, College of Animal Sciences, Zhejiang University, Hangzhou, PR China; 4 Center for Molecular Immunology, Institute of Microbiology, Chinese Academy of Sciences, Beijing, PR China

## Abstract

Bacille Calmette-Guérin (BCG) is an attenuated strain of *Mycobacterium bovis *currently used as a vaccine against tuberculosis. Global distribution and propagation of BCG has contributed to the *in vitro *evolution of the vaccine strain and is thought to partially account for the different outcomes of BCG vaccine trials. Previous efforts by several molecular techniques effectively identified large sequence polymorphisms among BCG daughter strains, but lacked the resolution to identify smaller changes. In this study, we have used a NimbleGen tiling array for whole genome comparison of 13 BCG strains. Using this approach, in tandem with DNA resequencing, we have identified six novel large sequence polymorphisms including four deletions and two duplications in specific BCG strains. Moreover, we have uncovered various polymorphisms in the *phoP-phoR *locus. Importantly, these polymorphisms affect genes encoding established virulence factors including cell wall complex lipids, ESX secretion systems, and the PhoP-PhoR two-component system. Our study demonstrates that major virulence factors are different among BCG strains, which provide molecular mechanisms for important vaccine phenotypes including adverse effect profile, tuberculin reactivity and protective efficacy. These findings have important implications for the development of a new generation of vaccines.

## Background

Bacille Calmette-Guérin (BCG) is an attenuated strain of *Mycobacterium bovis *and is the only available vaccine against tuberculosis (TB). Since 1974, BCG vaccination has been included in the World Health Organization (WHO) Expanded Program on Immunization. It is estimated that more than 3 billion individuals have been immunized with BCG and over 100 million doses of BCG are administered annually. Multiple studies have confirmed that BCG is generally safe and can protect children against disseminated disease, including tuberculosis meningitis [1,2]. BCG also provides cross-protection against leprosy [3]. However, the success of BCG against pulmonary TB in adults is still debated, since randomized clinical trials have reported protection efficacy ranging from 0–80% [4,5]. Several hypotheses for the variation in observed efficacy have been proposed [6-9].

One explanation concerns the heterogeneity of the BCG strains [6]. The original BCG was derived from a virulent strain of *M. bovis *isolated from a cow. From 1908 through 1921, this isolate was subjected to 230 passages on glycerinated potato bile medium, which generated an attenuated strain termed BCG [10]. Distribution and widespread use of BCG started around 1924 and was accompanied by changes in the manufacturing process in production facilities. For instance, while BCG in Sweden was transferred without interruption from bile potato to bile potato medium in accordance with Calmette's original practice [11], BCG production in Denmark involved alternating rounds of growth on potato bile medium and Sauton broth until 1949 when it was grown exclusively in Sauton medium [12]. Prior to the establishment of seed stocks in the 1960s, BCG was passaged continuously, and the changes in media and transfer schedules contributed to the "*in vitro *evolution" of BCG [6]. It is estimated that as many as 49 production substrains have been used at one time or another in various parts of the world [13], including the four major BCG vaccines in current use (BCG-Pasteur, -Danish, -Glaxo, and -Japan) [14]. The relative protective efficacy of BCG substrains is currently unknown [6,15].

Anecdotal reports have long indicated that BCG substrains exhibit phenotypic differences in growth characteristics, biochemical activities, ability to protect against challenge with *Mycobacterium tuberculosis *(*M. tb*), and residual virulence [16]. Over the past decade, numerous groups have sought to identify the genomic changes responsible for these phenotypes. The earliest whole genome comparisons confirmed that BCG was indeed related to, but distinct from *M. tb *and *M. bovis *[17-19]. Subsequent analyses of multiple vaccine strains have uncovered extensive genome diversity including both deletions and duplications in BCG substrains [18,20-22]. The phylogeny established by these molecular methods is consistent with the historical records of BCG dissemination [20,23,24]. For example, BCG strains acquired after 1927 exhibit the RD2 deletion, while nRD18 is only deleted in strains obtained after 1933. Other genomic changes are exclusive to individual daughter strains, and are associated with vaccine production at specific locations [22,24].

A number of molecular techniques have been used to investigate genomic polymorphisms in BCG strains. Early efforts using subtractive hybridization [18] and spotted oligonucleotide arrays [20,22,25] effectively identified large sequence polymorphisms, but lacked the resolution to identify smaller changes. More recently, complete genome sequencing has enabled high-resolution analysis of BCG-Pasteur 1173P2 [24], but sequences for other BCG lineages have yet to be determined. To identify potential genomic polymorphisms in other BCG substrains, we have employed a tiling array platform developed by NimbleGen Systems. This DNA microarray-based comparative genome sequencing technique allows high resolution detections of sequence polymorphisms [26-28]. Using this technique, in tandem with DNA resequencing, we have identified a number of novel genomic polymorphisms in BCG strains. Importantly, these polymorphisms affect genes that are known virulence factors and are expected to have a major impact on the immunogenicity and efficacy of individual vaccine strains.

## Results

We have used NimbleGen tiling arrays to analyze the genomic variability of 13 BCG strains, including BCG-Russia, -Japan, -Moreau, -Sweden, -Birkhaug, -China, -Prague, -Glaxo, -Danish, -Tice, -Phipps, -Frappier and -Pasteur. All of these strains, except BCG-China, have previously been subjected to genomic analysis by other methods [18,20,22,24,25]. The complete genome sequence of BCG-Pasteur 1173P2 is available [24]. The same BCG-Pasteur strain was included in the analysis to serve as an internal control for our experiments in addition to validating the NimbleGen technique. In each experiment, genomic DNA from *M. tb *H37Rv [29] acted as the common referent.

### Deletions and Duplications

A total of 42 deletions were identified. Twenty-five of these have been described previously [18,20,22,24,25]. Thirteen more represent transposons (e.g., IS*6110*) present in the referent strain (*M. tb *H37Rv), but absent from the *M. bovis *and BCG lineages [24,29,30]. Six duplications were identified, four (DU1, DU2-I, -II, -III) of which have been described previously [21,24]. These results confirm the validity of our approach, and the utility of tiling arrays for comparative genomics. A total of 4 novel deletions and 2 duplications were identified in our analysis. These novel deletions and duplications are described below.

Two deletions specific to BCG-Moreau were identified. The first is a 975 bp deletion (Table 1) that eliminates the distal end of *fadD26 *(*Rv2930/BCG2952*) and the start of *ppsA *(*Rv2931/BCG2953*). These genes are part of the genetic locus required for the biosynthesis of phthiocerol dimycocerosates (PDIMs) and phenolic glycolipids (PGLs) [31], two cell wall lipids known to be important for the virulence of *M. tb *and *M. bovis *[32-34]. In previous work, we demonstrated that BCG-Moreau does not produce PDIMs or PGLs [35], which is now explained by the *fadD26-ppsA *deletion identified in the current study.

**Table 1:** Novel deletions and duplications determined in current study.

**BCG strains**	**Polymorphisms**	**Start**	**End**	****Size (bp)****	**Genes affected**
Moreau	Deletion	3244503	3245478	975	*fadD26*, *ppsA*
		4370517	4371645	1128	Rv3887c
Birkhaug/Sweden	Deletion	1158377	1158622	245	*trcR*
		3834822	3834932	110	*whiB3*
Tice	Duplication	2017525	2039587	22062	*Rv1782-Rv1800*
Birkhaug	Duplication	1 4402007	20813 4411395	30201	*Rv3913-Rv0017c*

The coordinates correspond to the genome of *M. tb *H37Rv. The two deletions and DU-Tice were confirmed by PCR application and DNA sequencing.

The second novel polymorphism in BCG-Moreau is an 1128 bp deletion within *Rv3887c*/*BCG3942c *(Table 1). Although intact in other BCG substrains, this region overlaps with a 2.4-kb deletion (termed RDpan) found in some *M. bovis *strains [36], including the sequenced strain, AF2122/97 [30]. The *Rv3887c*/*BCG3942c *gene encodes a membrane transport protein and is part of the ESX-2 type VII secretion system [37]. The role of the ESX-2 system in virulence is unknown considering its variable presence among clinical *M. bovis *isolates from both France and England [36]. However, loss of the Rv3887c membrane transporter likely eliminates the secretion of ESAT-6- and CFP-10-like antigens [37] and influences the immunogenicity of the vaccine strain.

Two novel deletions were identified in BCG-Sweden and BCG-Birkhaug. These polymorphisms are identical between the two BCG strains, which is consistent with their genealogy [24]. The first deletion comprises 110 bp and disrupts the promoter and translational start site of *whiB3 *(*Rv3416/BCG3486*) [see Additional file 1]. The other is a 245 bp deletion within *trcR *(*Rv1033c/BCG1091c*) (Table 1). Both genes encode transcriptional regulators known to impact virulence.

WhiB3 belongs to a family of seven *M. tb *transcriptional regulatory proteins that contain iron-sulfur clusters and are predicted to regulate gene expression in response to environmental stimuli [38]. WhiB3 responds to oxygen and nitric oxide, and is important for regulation of carbon metabolism [39]. The deletion of *whiB3 *in *M. bovis *attenuates *in vivo *growth in guinea pigs [40]. TrcR is the response regulator of the TrcR-TrcS two-component system. Deletion of *trcS *from *M. tb *generates a hypervirulent phenotype such that the strain exhibits increased lethality in SCID mice [41].

**Figure 1:**
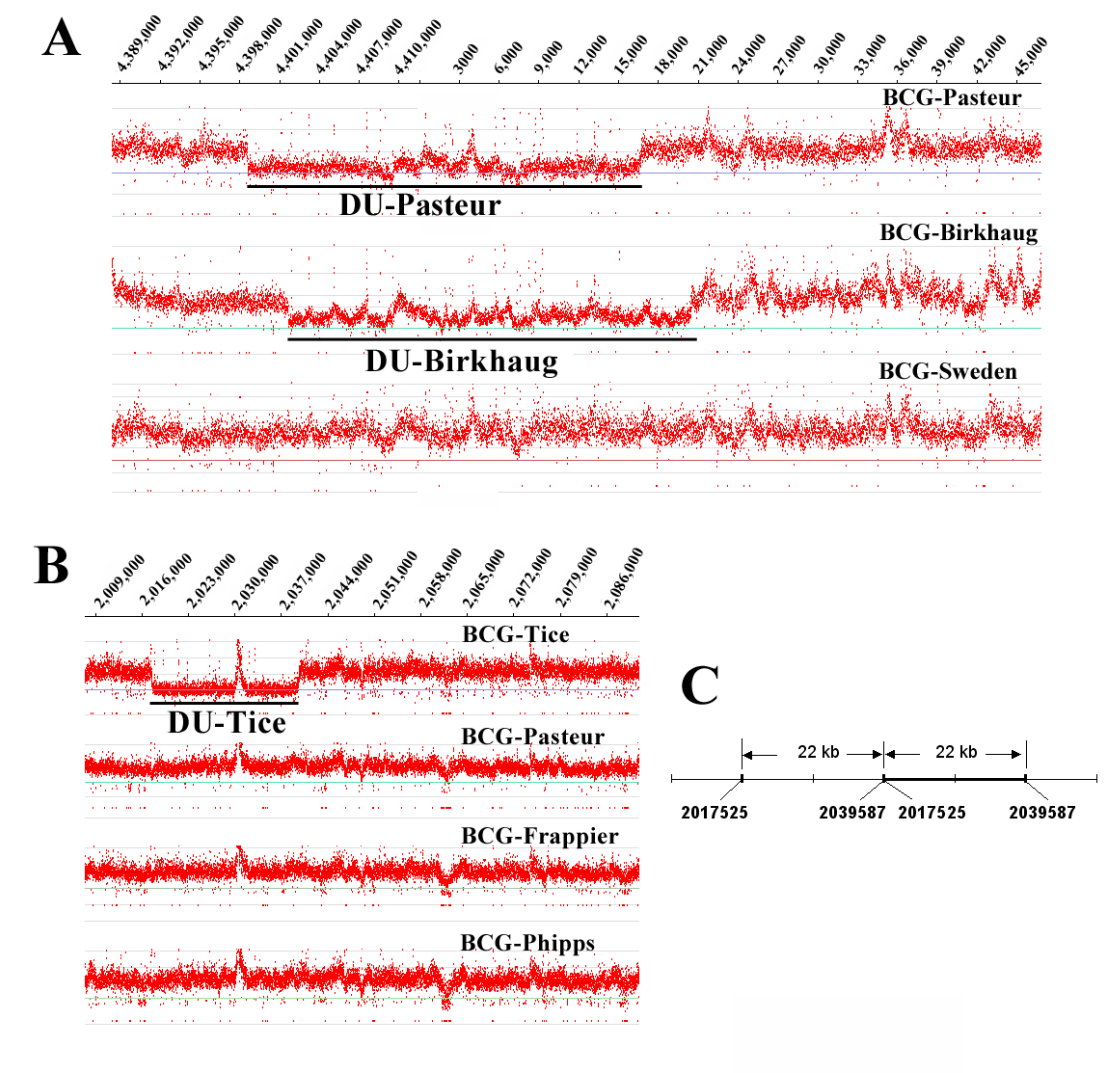
**Novel duplications identified in BCG-Birkhaug and BCG-Tice by NimbleGen tiling array**. Sections of the ratio plot are shown. The ratio of the reference (*M. tb *H37Rv) probe intensity (Cy5) was divided by the test (BCG strain) probe intensity (Cy3). Reference probes and test probes that do not span a mutation should represent full-length perfect match hybridization, and thus should have similar intensities, with a reference/test ratio near 1. If the test genome contains an amplification event (increased copy number when compared to the reference), then the reference/test ratio will shift below 1. (**A**) Novel duplication (DU-Birkhaug) identified in BCG-Birkhaug, which is analogous to the DU-Pasteur (DU1) but has different borders. The same genomic region of BCG-Sweden, which is closely related to BCG-Birkhaug, is shown for comparison. (**B**) Novel duplication (DU-Tice) identified in BCG-Tice. Three other BCG strains belonging to the same group (DU2-IV) are shown for comparison. (**C**) The precise border of DU-Tice is mapped by PCR amplification using primers specific to the junction. The two copies are immediately adjacent to each other and overlap by 1 bp.

Although the genomic profiles of BCG-Birkhaug and BCG-Sweden are similar, we have also found that BCG-Birkhaug is distinguished by a strain-specific duplication, named DU-Birkhaug. This spans the origin of replication and is analogous to the DU1 duplication in BCG-Pasteur [21,24] (Fig. 1A). However, the borders of the DU-Birkhaug are different. Whereas DU1 encompasses 29.6 kb from *Rv3910 *to *pknB*/*Rv0014*, DU-Birkhaug spans a slightly different region, from *trxB*/*Rv3913 *to *rodA/Rv0017c*. Most of the genes in these regions are involved with DNA replication and cell division. Unlike DU1, DU-Birkhaug also appears to be in a genomic location distant to its original copy. Initial PCR-based attempts to characterize the boundaries of this duplication assumed that the second copy was nearby failed to detect a product (data not shown). As such, the genome location of DU-Birkhaug remains unknown.

Our analysis also revealed a novel duplication in the genome of BCG-Tice termed DU-Tice. It comprises a 22-kb duplication that encompasses *Rv1782-Rv1800 *(Fig. 1B). The precise boundaries and location of this duplication were determined using primers at the junction (Fig. 1C). Interestingly, DU-Tice encodes the ESX-5 secretion system [37,42]. This includes several conserved membrane transporters (*Rv1782*, *Rv1783*, *Rv1795*, and *Rv1797*), a membrane associated ATPase (*Rv1784*), a set of PE/PPE genes (*Rv1787-Rv1792*) and the ESAT-6 and CFP-10 family proteins (*esxM *and *esxN*) [37]. ESX-5 is absent from the genome of the fast-growing, non-pathogenic *M. smegmatis*, but present in both the *M. avium *complex and *M. marinum*. The role of ESX-5 in virulence has been demonstrated in *M. marinum *[37,42]. It has been suggested that the ESX clusters evolved via gene duplication [43] and DU-Tice offers the first snapshot of such an event.

To our knowledge, we have conducted the first genomic analysis of BCG-China, which is a descendant of BCG-Danish obtained from the Statens Serum Institut around 1947. Consistently, BCG-China exhibits the DU2-III duplication and deletion of RD2 (data not shown), which is similar to other BCG-Danish derivatives, including BCG-Prague (obtained in 1946 from passage 725) [44], BCG-Glaxo (obtained in 1954, from passage 1077) and BCG-Danish (lyophilized in 1961, from passage 1331) [45]. However, BCG-China and -Prague do not contain the previously described deletion of *Rv1810*, which is characteristic of BCG-Glaxo and -Danish. As such, the *Rv1810 *deletion must have occurred between 1947 and 1954. Coincidentally, this period corresponds to the replacement of potato bile medium by Sauton medium for BCG production in Denmark [12].

### Polymorphisms of *phoP-phoR*

The PhoP-PhoR system is one of the 11 two-component systems found in the *M. tb *genome [29]. The PhoR protein is a transmembrane histidine kinase that transmits signals from the environment by autophosphorylation. The phosphoryl group is then transferred to PhoP, a response regulator that regulates the expression of multiple genes [46]. Recently, several studies have demonstrated that the PhoP-PhoR system, particularly PhoP, plays an essential role in *M. tb *virulence [26,46-48]. A single point mutation (S219L) in the DNA binding region of PhoP partially accounts for the attenuation of the H37Ra strain of *M. tb *[26]. Furthermore, a *phoP *mutant of *M. tb *was found to be more attenuated than BCG-Pasteur in SCID mice infections [47]. Our NimbleGen analysis revealed some weak signals in the *phoP-phoR *region (not shown), which prompted us to resequence these genes. The DNA fragment containing the promoter region of *phoP*, the ORFs of *phoP *and *phoR*, and the intergenic region was PCR amplified from each BCG strain and determined by DNA sequencing.

**Figure 2:**
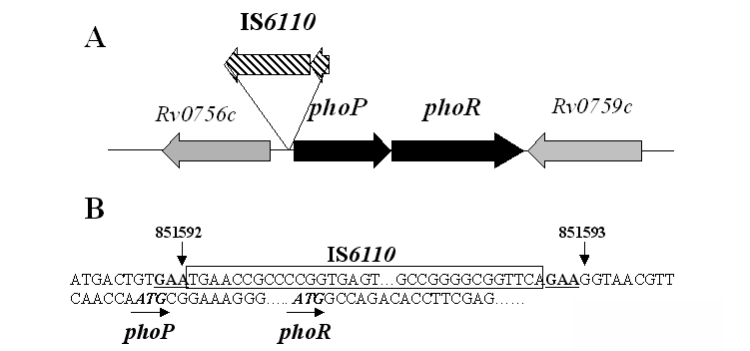
**IS*6110 *insertion in the *phoP *promoter in BCG-Russia, -Moreau, and -Japan**. (**A**) Schematic representation of the *phoP-phoR *locus with IS*6110 *inserted in an inverse orientation 18 bp upstream from *phoP *start codon. (**B**) Nucleotide sequence surrounding IS*6110*. The IS*6110 *sequence is boxed. The GAA direct repeats flanking the IS*6110 *insertion site is underlined and in boldface. The ATG start codons of *phoP *and *phoR *are indicated by arrows and in boldface.

Our sequence analysis revealed a number of polymorphisms in the *phoP-phoR *locus in various BCG strains compared to the genome sequence of *M. bovis*. The three early BCG substrains, BCG-Russia, -Japan, and -Moreau, contain an identical IS*6110 *(1,356 bp) insertion at nucleotide 851593 of the *M. tb *genome, which is 18 bp upstream of the start codon of *phoP *(Fig. 2). This IS*6110 *element is identical to many other copies of IS*6110 *found in various locations in the *M. tb *genome. It is flanked by a 3-bp direct repeat (GAA) on both sides and is in an inverse orientation of *phoP-phoR *(Fig. 2). The presence of an IS*6110 *element in the promoter region of *phoP *in BCG-Russia, -Japan, and -Moreau has been described previously, but its insertion site and orientation were not determined until now [49]. Although not present in *M. tb *H37Rv or *M. bovis *AF2122/97, an IS*6110 *insertion in the *phoP *promoter was found in a clinical strain of *M. bovis *termed B strain, which was responsible for a severe nosocomial outbreak of multidrug resistant TB in humans in Spain [50,51]. However, unlike the three BCG strains, the IS*6110 *insertion in the *M. bovis *B strain is located at 75 bp upstream of the start codon of *phoP *and is in the same orientation as *phoP-phoR *[50]. The potential effect of IS*6110 *on *phoP *expression is described in the 'Discussion' section.

Three other novel *phoP-phoR *polymorphisms that likely impact their functions were also uncovered by our sequencing analysis. An identical, 11-bp deletion within the ORF of *phoR *was uncovered in BCG-Sweden and BCG-Birkhaug (ACCGGACTGGG, nucleotides from 853689 to 853699, *M. tb *genome coordinates). This deletion changes the amino acid sequence of 54 residues (residues 432 to 485) in the C-terminal of PhoR. This polymorphism is different than the previously described 10 bp deletion within *phoR *present in BCG-Danish and BCG-Glaxo, which affects residues 91–485 [24]. BCG-Frappier also contains a single nucleotide deletion (A at 852701, *M. tb *genome coordinates), causing a frame-shift mutation that affect residues 103–485 of PhoR. Together, these results indicate that besides BCG-Danish and BCG-Glaxo, BCG-Sweden, -Birkhaug, and -Frappier also contain a defective *phoR *gene.

A single nucleotide insertion within the ORF of *phoP *was uncovered in BCG-Prague (G, between nucleotides 852067 and 852068, *M. tb *genome coordinates) [see Additional file 2]. This frame shift mutation changes the C-terminal sequence (residues 154–247) of PhoP, which is the DNA binding domain (residues 144–247) [52-54]. As such, BCG-Prague is a natural *phoP *mutant.

Single point mutations in PhoP or PhoR are also found in various BCG strains and are summarized in Table 2. In contrast, sequences of the *phoP-phoR *locus of BCG-Phipps, -Tice, and -Pasteur are identical to the published sequence of BCG-Pasteur and *M. bovis *[24,30].

**Table 2:** Novel polymorphisms of *phoP-phoR *in BCG strains identified in current study.

**BCG strains**	**Polymorphisms of *phoP***	**Predicted effects on PhoP**	**Polymorphisms *phoR***	**Predicted effects on PhoR**
Russia Japan Moreau	IS*6110 *insertion at 18 bp upstream of ATG start codon	Upregulation of *phoP*	One SNP in BCG-Moreau. No SNP in BCG-Russia or -Japan	D322G mutant in BCG-Moreau
Birkhaug Sweden	ND	NA	11 bp deletion within ORF	Mutated 54 residues of the C-terminal
Prague	Single base insertion within ORF	Mutated 94 residues of the C-terminal	ND	NA
Frappier	SNP within ORF	T9M mutant	Single base deletion within ORF	Mutated 383 residues of the C-terminal
China	SNP within ORF	P151S mutant	ND	NA

The sequences of *phoP-phoR *in BCG-Phipps, BCG-Tice, and BCG-Pasteur are 100% identical to the published genome sequences of *M. bovis *AF2122/97 [30]. BCG-Danish and -Glaxo contains a 10 bp deletion within *phoR*, which was described previously [24]. ND: polymorphisms not detected. NA: no affect.

## Discussion

**Figure 3:**
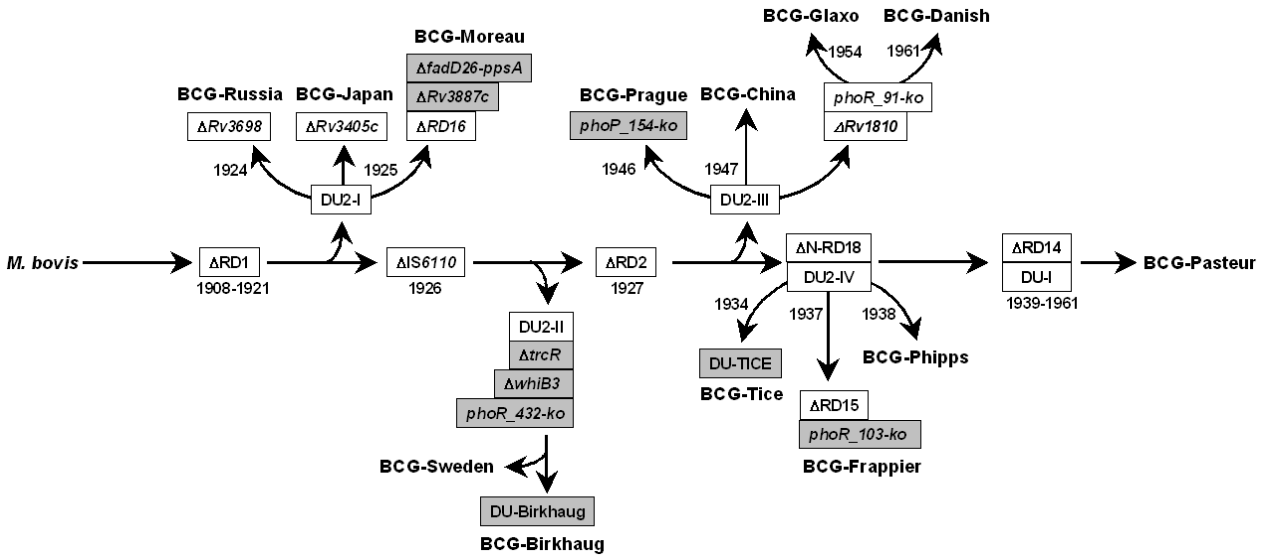
**Refined genealogy of BCG vaccines**. The genealogy is modified from a previous model [24]. Genetic markers identified in this work are highlighted.

The loss of the RD1-encoded ESX-1 protein secretion system during 1908–1921 contributes to the attenuation of BCG ([55], see also Fig. 3). However, because reintroduction of ESX-1 into BCG does not restore full virulence, other genetic lesions are also involved [56]. Whole genome sequence comparison reveals 2,223 single nucleotide polymorphisms (SNPs) between BCG-Pasteur and *M. tb *H37Rv, and 736 SNPs between BCG-Pasteur and *M. bovis *AF2122/97 [24]. NimbleGen analysis revealed 1,010 SNPs between BCG-Pasteur and *M. tb *H37Rv. Of which, 945 SNPs were correctly identified when comparing to the complete genome sequence of BCG-Pasteur. Thus the NimbleGen technique has a limited ability to detect SNPs but the majority of identified SNPs are accurate. Our NimbleGen analysis also revealed numerous SNPs (ranging from ~400 to ~1,800) between individual BCG strains and *M. tb *H37Rv, and the majority of changes detected by the NimbleGen technique are also present in BCG-Pasteur (data not shown). This suggests that the loss of RD1 and the accumulation of a number of point mutations during the 230 passages *in vitro *likely account for the initial loss of virulence by 1921. Subsequent dissemination of BCG to various parts of the world, accompanied by changes in the manufacturing process, further affected the residual virulence and immunogenicity of individual BCG strains. As such, some strains are more virulent than others in animal models of infection [57] and also exhibit differential ability to induce adverse reactions (reactogenicity) following vaccination in neonates [58]. Our current work begins to provide some explanation for these observed differences (Fig. 3).

Consistent with a previous study [58], we find that the earliest distributed BCG strains, BCG-Russia, -Japan, and -Moreau, all contain a second copy of IS*6110 *that is inserted in the promoter region of *phoP*. A similar, albeit distinct, insertion of IS*6110 *in the *phoP *promoter was also found in a virulent strain called *M. bovis *B strain [50]. The presence of IS*6110*, which is in the same orientation as *phoP*, increases the expression of *phoP *and (the resulting increase in virulence) was thought to be responsible for the outbreak of *M. bovis *B strain in humans [50]. Similarly, the expression level of *phoP *was found to be higher in BCG-Japan than in BCG-Pasteur [24]. However, in BCG-Japan, -Russia, and -Moreau, the IS*6110 *is in the inverse orientation of *phoP *(Fig. 2). As such, how IS*6110 *upregulates *phoP *expression in BCG is not immediately apparent and likely involves a different mechanism. One possibility is the elimination of *phoP *autoregulation. It was shown that PhoP protein, albeit from H37Ra, binds to three 9-bp direct repeats within the *phoP *promoter sequence and represses its own expression [52]. In BCG, IS*6110 *is inserted between the PhoP binding sites and the start codon of *phoP*, which could impair the repression by PhoP and subsequently increase *phoP *expression. Alternatively, an unidentified promoter sequence within IS*6110 *in the same orientation of *phoP *could drive the expression of *phoP*. The presence of the second copy of IS*6110 *in these early BCG strains also suggests that the original BCG isolated in 1921 might have been derived from a highly virulent *M. bovis *strain containing the same IS*6110 *element. This IS*6110 *was subsequently lost in other BCG strains (Fig. 3) and is not present in most clinical strains of *M. bovis *and *M. tb *isolated in modern times [50,59]. Given the important role of PhoP in *M. tb *virulence, higher expression of *phoP *could explain why BCG-Russia is generally considered more virulent than other BCG strains [6]. However, in the other early strains, BCG-Moreau and BCG-Japan, the loss of lipid virulence factors PDIMs and PGLs appears to have a more pronounced effect on virulence. Consequently, these two strains, together with BCG-Glaxo, which also lacks PDIMs and PGLs, and as we have described previously, are more attenuated and less reactogenic than other BCG strains [35]. The deletion of *fadD26-ppsA *described here provides a genetic mechanism for the defective PDIM/PGL biosynthesis in BCG-Moreau. However, this region is intact in BCG-Japan and BCG-Glaxo, indicating that other mechanisms may also lead to the PDIM/PGL defect.

BCG-Sweden was obtained from the Institut Pasteur in 1926 while Konrad Birkhaug acquired the strain that bears his name around 1927 [6]. Previous studies indicated that these strains differ from other early BCG strains (i.e., BCG-Russia, -Japan, -Moreau) only by the loss of the IS*6110 *element described above. Our current work reveals three novel deletions shared by BCG-Sweden and BCG-Birkhaug (Fig. 3), which distinguish them from other early strains. Two deletions affect the regulatory proteins WhiB3 and TrcR, and have different impacts on virulence. The *whiB3 *gene appears to be important for virulence. The *M. bovis whiB3 *mutant is attenuated for growth in guinea pigs but not in mice [40]. Conversely, the *trcRS *two-component system has a negative impact on virulence. Deletion of *trcS *from *M. tb *generates a hypervirulent phenotype in SCID mice [41]. BCG-Sweden was used in Sweden from 1926 until 1978 and was then replaced by BCG-Danish because of the high frequency of osteitis associated with the former strain [60]. The deletion of *trcR *may contribute to the reactogenicity of BCG-Sweden.

The other deletion found in BCG-Sweden and BCG-Birkhaug affects the *phoR *gene of the *phoP-phoR *two-component system. Remarkably, three other late BCG strains, BCG-Danish, -Glaxo, and -Frappier also contain a defective *phoR *gene. Together a total of five BCG strains are natural *phoR *mutants. However, three distinct mutations are found among these five strains, which correspond to their genealogy (Fig. 3). The role of *phoR *in virulence is less understood than for *phoP*. Among its many functions, *phoP *is required for the biosynthesis of trehalose-containing cell wall lipids [48,61,62]. Contrastingly, *phoR *does not seem to be required for this function [62]. Nevertheless, the fact that the *phoR *mutation has been acquired by different groups of BCG strains by three independent events and genetic mechanisms suggests that there was a common selective pressure and an important role for this gene during the *in vitro *evolution of BCG.

Another BCG strain that contains a major mutation in the *phoP-phoR *system is BCG-Prague. A single nucleotide insertion in the ORF of *phoP *changes the C-terminal sequence, which contains the DNA binding domain of PhoP [52-54]. As such, BCG-Prague is a natural *phoP *mutant and likely to be more attenuated than other BCG strains. This is consistent with the study by Lagranderie *et al.*, which showed in mice models of infection that BCG-Prague exhibited more attenuated phenotypes compared to three other BCG strains (BCG-Russia, -Pasteur, and -Glaxo) [57]. Compared to 11 other BCG strains, including BCG-Russia, -Moreau, -Japan, -Sweden, -Danish, -Glaxo, and -Pasteur that have been analyzed in the current study, BCG-Prague consistently exhibited the weakest ability to induce delayed type hypersensitivity to tuberculin in children [63] or in guinea pig models [64]. Because of the traditional presumption that tuberculin reactivity is associated with vaccine potency, BCG-Prague, which was used in Czechoslovakia between 1951–1980 and appeared to be effective, was replaced by BCG-Russia in 1981 [58]. An immediate increase of BCG-induced osteitis cases was observed in Czechoslovakia following the switch of BCG-Prague to BCG-Russia [65]. The *phoP *mutation detected in the current study may explain the weak tuberculin sensitivity induced by BCG-Prague. It was recently shown that a *phoP *mutant of *M. tb *was more attenuated than BCG-Pasteur and confers an equivalent protection in mice against *M. tb *challenge. In the guinea pig model, the *M. tb phoP *mutant showed superior protection to BCG-Pasteur against a high dose challenge with *M. tb *[47]. Consequently, the *M. tb phoP *mutant is now being evaluated as a vaccine candidate to replace BCG [66]. Since BCG-Pasteur contains an intact *phoP *gene, and in light of our finding, it would be worthy to compare the *M. tb phoP *mutant with BCG-Prague in terms of safety and protective efficacy.

The novel duplication uncovered in BCG-Tice (DU-Tice) may have an impact on its residual virulence and immunogenicity. DU-Tice contains the entire ESX-5 secretion system, which is one of the five type VII secretion systems found in the *M. tb *complex [37]. Importantly, besides the RD1-encoded ESX-1, ESX-5 is the only other ESX system that has been shown to be involved in virulence thus far [37]. ESX-5 is conserved in other pathogenic mycobacteria and reported to facilitate the cell-to-cell spread of *M. marinum *in infected macrophages, a function shared by ESX-1 [42]. However, ESX-5 does not complement the loss of virulence caused by ESX-1 deletion, suggesting that they play distinct roles in virulence [37]. Horwitz and co-workers have used BCG-Tice as the host strain to overexpress antigen 85B. This resulted in a recombinant strain termed rBCG30 that exhibits superior protective efficacy over BCG-Tice and is currently being evaluated as a vaccine candidate in human clinical trials [67-71]. The rBCG30 Tice strain also showed significantly stronger immune response and better protection against *M. tb *challenge than the rBCG30 strain based on BCG-Connaught [69]. The duplication of ESX-5 in BCG-Tice, which could increase the residual virulence and immunogenicity, may partially account for the benefit associated with rBCG30 Tice.

## Conclusion

Our current work has uncovered six large sequence polymorphisms not described previously, including two deletions exclusive to BCG-Moreau, two deletions shared by BCG-Sweden and BCG-Birkhaug, as well as the DU-Birkhaug and DU-Tice duplications. Moreover, we have uncovered a number of polymorphisms in the *phoP-phoR *locus in various BCG strains. Remarkably, these polymorphisms affect genes that are well known to have major impact on the virulence of *M. tb *or *M. bovis*. These include genes involved in the biosynthesis of lipid virulence factors PDIMs/PGLs, genes that encode the ESX family type VII secretion system, and the *phoP-phoR *two-component regulatory system. As such, the current collection of BCG comprises natural mutants of established virulence factors identified thus far. BCG-Moreau, -Japan, and -Glaxo are PDIMs/PGLs deficient mutants. BCG-Prague is a *phoP *mutant. BCG-Sweden, -Birkhaug, -Danish, -Glaxo, and -Frappier are defective in *phoR*. BCG-Sweden and BCG-Birkhaug are also *whiB3 *mutants. These findings have important implications on the current effort and future development of TB vaccines. Currently, among major efforts, a *phoP *mutant of *M. tb *and several recombinant BCG strains including rBCG30 Tice, rBCG-Aeras 403 Danish, rBCGΔ Ure::CHly Pasteur, BCG::RD1 Pasteur, and rBCGΔ Sod Tice are being evaluated as new generation TB vaccines in preclinical or clinical trial studies [72]. In addition, a mutant of *M. bovis *deficient in PDIMs/PGLs is being considered as a vaccine to protect wildlife against bovine tuberculosis [73]. Our previous study [35] and current work provide direct evidence that BCG vaccine strains are different in major virulence factors, and likely have different vaccination properties including safety, immunogenicity, and efficacy. Since new vaccine candidates are evaluated for their vaccination properties relative to BCG, the appropriate choice of BCG strain for these studies is critical. Furthermore, because it is likely that BCG will continue to play a role in tuberculosis control by being included in forthcoming clinical trials, as either a primer to be boosted by new components (e.g. subunit or DNA vaccine) or as an integral component (e.g. recombinant BCG) of new vaccines, greater attention must be given to the benefits that a particular strain may – or may not – offer.

## Methods

### Bacterial strains

The mycobacterial strains used in this study were: *Mycobacterium tuberculosis *H37Rv, BCG-Russia (ATCC 35740), BCG-Moreau/Rio de Janeiro, BCG-Japan, BCG-Sweden, BCG-Birkhaug (ATCC 35731), BCG-Denmark 1331 (ATCC 35733), BCG-China, BCG-Prague, BCG-Glaxo (ATCC 35741), BCG-Tice (ATCC 35743), BCG-Frappier (ATCC 35735), BCG-Connaught, BCG-Phipps (ATCC 35744), and BCG-Pasteur 1173. All BCG strains except BCG-China have been previously described [20]. BCG-China was obtained from Shanghai Institute of Biological Product, which is the main manufacturer for BCG in China.

### Genomic DNA extraction and labeling

Mycobacteria were cultured in Middlebrook 7H9 broth supplemented with 10% ADC. Chromosomal DNA was extracted using QIAGEN Genomic-tip 100/G kit (Qiagen) and then labeled with a random primer reaction. DNA (1 μg) was mixed with 1 O.D. of 5'-fluorescence dye labeled random nonamer (Cy3 for BCG strains and Cy5 for reference strain H37Rv) (TriLink Biotechnologies) in 62.5 mM Tris-HCl, 6.25 mM MgCl_2 _and 0.0875% ß-mercaptoethanol, denatured at 98°C for 5 min, chilled on ice, and incubated with 100 units Klenow fragment (NEB) and dNTP mix (6 mM each in TE) for 2 h at 37°C. Reactions were terminated with 0.5 M EDTA (pH 8.0), precipitated with isopropanol, and resuspended in water. A fifty-fold amplification was typically achieved.

### Design of mutation mapping microarray

Mutation mapping microarrays were designed with NimbleGen algorithms that select a 29-mer oligonucleotide every 7 bases on each strand of the reference genome sequence (Genbank Accession AL123456) [29]. All probes were synthesized in parallel on a four-array set using a Digital Light Processor™ (Texas Instruments, Plano Texas) and photoprotected by phosphoramidite chemistry (Maskless Array Synthesis) (NimbleGen Systems, Madison WI) in a random probe layout [74,75].

### Microarray hybridization

Labeled genomic DNA was hybridized to arrays in the NimbleGen Hybridization Buffer at 42°C for 16 hr using a MAUI hybridization system (BioMicro Systems, Inc. Salt Lake City, Utah). Labeled genomic DNA (5 μg) from the reference strain *M. tb *H37Rv and from each BCG strain were co-hybridized to each array. Arrays were washed with NimbleGen wash buffer, and were then spun dry in a microarray high-speed centrifuge (TeleChem International, Inc., Sunnyvale, CA) and stored until scanned.

### Analysis of mapping array data and design and hybridization of resequencing microarrays

Microarrays were scanned at 5 μm resolution using the Genepix^® ^4000B scanner (Axon Instruments, Union City CA), and pixel intensities were extracted using NimbleScan™ v2.4 software (NimbleGen). Probes that spanned potential mutations were identified by NimbleGen software. Probe sequences corresponding to all possible candidate mutation sites were selected for resequencing. The strategy that was used to automatically generate the sequencing array is similar to that described previously [28]. Briefly, 8 probes per base position were analyzed, 4 per genome strand. These probes contain all possible alleles at a centrally located position. The length, melting temperature and mismatch position of each probe were optimized. When target DNA is hybridized to these arrays the perfectly matched probe will hybridize more strongly than the three corresponding mismatched probes for each strand. The differential signal intensity between the perfectly matched probe and mismatched probes allows the base to be determined precisely. These resequencing arrays were synthesized, hybridized with labeled genomic DNA from each BCG strain and scanned as above. Sequence base assignments were made using a machine-learning algorithm [76]. Putative mutation-containing DNA segments were PCR amplified and verified by capillary sequencing [see Additional file 3]. The microarray data has been deposited in the Center for Information Biology Gene Expression Database (CIBEX; ), with the accession number of CBX70.

## Authors' contributions

ASL, VT, ZW, and XY performed the experiments and participated in data analysis. DCA participated in data analysis and co-authored the manuscript. GFG oversaw the experiments. BZ oversaw the experiments and participated in data analysis. JL oversaw the experiments, analyzed the data, and wrote the manuscript.

## Supplementary Material

Additional file 1 Reference (*M. tb *H37Rv) to test (BCG-Birkhaug) ratio plot showing the 110 bp deletion within *whiB3 *gene. Click here for file

Additional file 2 **DNA sequencing chromatograph showing the single nucleotide (G) insertion within the *phoP *gene in BCG-Prague. **This SNP was confirmed by repeating the PCR amplification and DNA sequencing. Click here for file

Additional file 3 DNA primers used for PCR amplification and DNA resequencing of identified novel polymorphisms. Click here for file
