# Supplementary figures and images for: Novel genome polymorphisms in BCG vaccine strains and impact on efficacy
Source: BMC Genomics. 2008 Sep 15;9:413. doi: 10.1186/1471-2164-9-413 (PMC2553098; doi:10.1186/1471-2164-9-413)

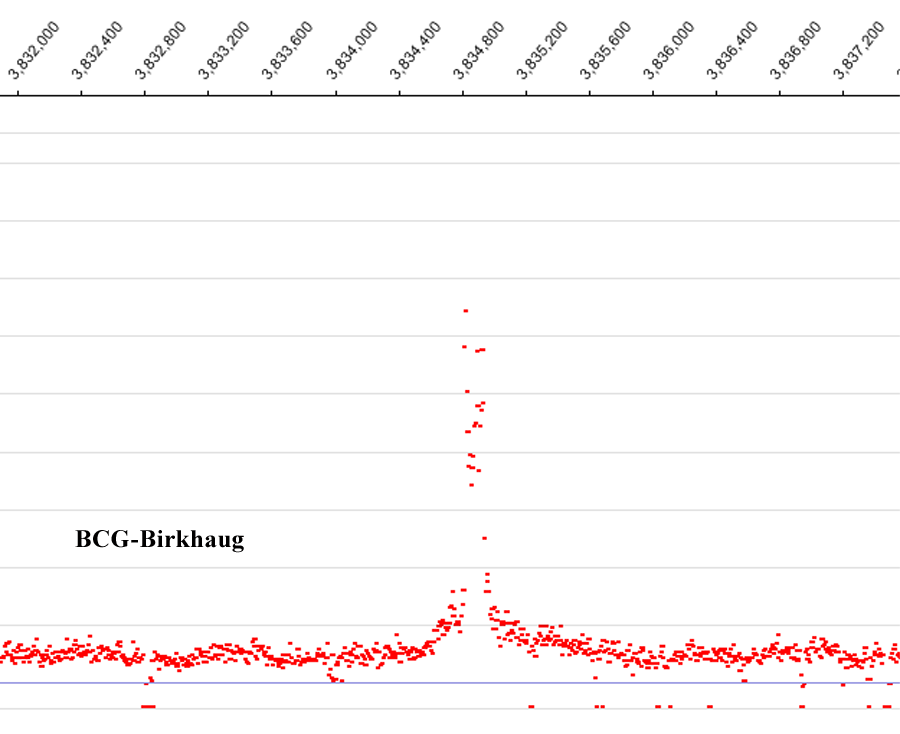

Supplement: Additional file 1 — Reference (M. tb H37Rv) to test (BCG-Birkhaug) ratio plot showing the 110 bp deletion within whiB3 gene. [file 1471-2164-9-413-S1.tiff]

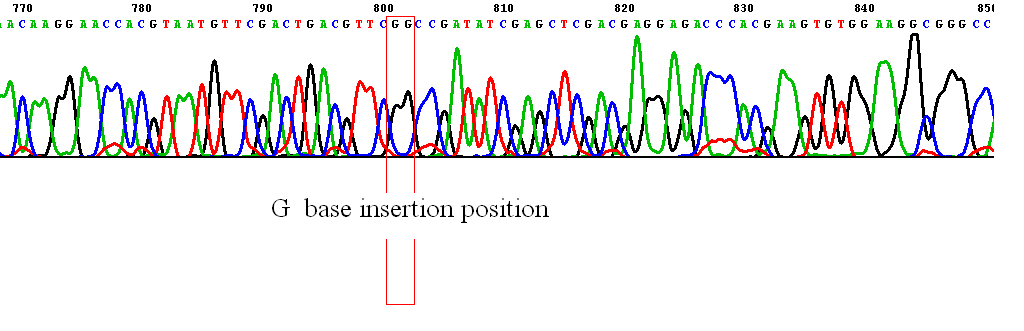

Supplement: Additional file 2 — DNA sequencing chromatograph showing the single nucleotide (G) insertion within the phoP gene in BCG-Prague. This SNP was confirmed by repeating the PCR amplification and DNA sequencing. [file 1471-2164-9-413-S2.tiff]
