# Supplementary material for: Novel genome polymorphisms in BCG vaccine strains and impact on efficacy
Source: BMC Genomics. 2008 Sep 15;9:413. doi: 10.1186/1471-2164-9-413 (PMC2553098; doi:10.1186/1471-2164-9-413)
Supplement: Additional file 3 — DNA primers used for PCR amplification and DNA resequencing of identified novel polymorphisms. [file 1471-2164-9-413-S3.doc]

| **Regions amplified** | **Forward primers** | **Reverse primers** |
| --- | --- | --- |
| *fadD26-ppsA* | 5'-ttccgaccgggactgtggt-3' | 5'-tgtgcagccgatattggtta-3' |
| *rv3887c* | 5'-ccctgggtgatgaagaggc-3' | 5'-gcggtcaacatttgctacga-3' |
| *trcR* | 5'-cctttccagatggctggag-3' | 5'-ggacttgagaggatacggg-3' |
| *whiB3* | 5'-gggatgacctgggcaaat-3' | 5'-ccgtcgggatggaagaac-3' |
| DU-Tice | 5'-gatgcacagcggcgtctga-3' | 5'-gtggctgaagagagccgcg-3' |
| DU-Birkhaug | 5'-ccaccaacccaagctcttc-3' | 5'-ctgatcgcggaagaagaatcc-3' |
| *phoP-phoR* | 5'-atcgtcgtccaatagcctg-3' | 5'-agtgccccacctacacatcc-3' |
